# A Systematic Literature Review of the Epidemiological, Diagnostic Workup, Humanistic, and Economic Burden of Alzheimer’s Disease in Spain

**DOI:** 10.7759/cureus.101581

**Published:** 2026-01-15

**Authors:** Miren Altuna, Eloy Rodríguez, Mercedes Núñez, Ángel Trueba-Saiz, Luis Lizan, Silvia Díaz-Cerezo

**Affiliations:** 1 Center for Research and Memory Clinic, Centro de Investigación y Terapias Avanzadas (CITA)Alzheimer Foundation, San Sebastián, ESP; 2 Department of Neurology, Bioaraba Health Research Institute, Araba University Hospital-Txagorritxu, Vitoria-Gasteiz, ESP; 3 Department of Medicine, Faculty of Health Sciences, University of Deusto, Bilbao, ESP; 4 Department of Neurology, Marqués de Valdecilla University Hospital, Santander, ESP; 5 Department of Neurodegenerative Diseases, Institute for Research Marqués de Valdecilla (IDIVAL), Santander, ESP; 6 Department of Medicine and Psychiatry, University of Cantabria, Santander, ESP; 7 Department of Research, Network Center for Biomedical Research in Neurodegenerative Diseases, National Institute of Health Carlos III, Madrid, ESP; 8 Department of Medical Affairs, Eli Lilly and Company, Madrid, ESP; 9 Department of Outcomes Research, Outcomes’10 a Product-Life Group Company, Castellon de la Plana, ESP

**Keywords:** alzheimer’s disease, caregiver burden, costs, early diagnosis, epidemiology

## Abstract

Introduction: Alzheimer’s disease (AD), the leading cause of dementia, affects over 700,000 individuals in Spain, with prevalence expected to rise due to population aging and improved diagnostic accuracy. Around 40,000 new cases are diagnosed annually, yet early-stage AD dementia remains underdiagnosed, limiting understanding of its full epidemiological, clinical, humanistic, and economic burden in Spain.

Methods: A systematic literature review (SLR) was conducted on the burden of AD dementia in Spain, focusing on observational studies published from January 2019 to January 2024. Searches were performed in PubMed/Medical Literature Analysis and Retrieval System Online (MEDLINE), Medicina en Español (MEDES), and Índice Bibliográfico Español en Ciencias de la Salud (IBECS), following Preferred Reporting Items for Systematic Reviews and Meta-Analyses (PRISMA) guidelines.

Results: Twenty-six publications were included, mainly addressing moderate-to-severe AD dementia, with limited evidence on mild cognitive impairment (MCI) due to AD. Commonly used tools included the Mini-Mental State Examination (MMSE), Barthel Index, and Global Deterioration Scale (GDS). Neurologists were the primary clinicians involved in diagnosis (78.6%), though etiological diagnosis often lacked core AD biomarkers. Most caregivers were informal (mainly women), experiencing significant declines in health-related quality of life (HRQoL). Non-healthcare costs accounted for 84.6%-90.7% of total expenditures, followed by direct healthcare (6.1%-10.0%) and social care costs (2.8%-4.6%). Indirect costs, mostly from reduced working hours, represented 0.5%-0.8%.

Conclusions: This study highlights the need to improve early diagnosis of AD dementia and to establish reliable health registries to measure its burden. The lack of evidence on practice heterogeneity hinders standardized care strategies. Addressing these gaps is essential to improve patient management, ensure equitable access to timely and accurate diagnosis, and facilitate access to emerging disease-modifying treatments for AD dementia.

## Introduction and background

Alzheimer’s disease (AD) is the leading cause of dementia, accounting for 60%-80% of all cases [1, 2]. It is a neurodegenerative disorder marked by progressive cognitive decline, mostly with amnesic symptoms [3], and its neuropathological signs include accumulation of beta-amyloid plaques and tau neurofibrillary tangles (NFT) in the brain [1, 2]. Over 90% of AD cases have a late onset, where symptoms develop at or after the age of 65 [4]. Consequently, AD is a leading cause of disability and dependence among the elderly [1], accounting for 11.9% of the years lived with disability due to chronic diseases [1]. The disease progresses over decades through several stages: preclinical (asymptomatic) stage, mild cognitive impairment (MCI), and mild, moderate, and severe dementia [1, 5]. Symptoms worsen over time, leading to a gradual decline in daily functionality and independence. Simple tasks, such as dressing or eating, become increasingly challenging until individuals with severe dementia often require full-time care and assistance [1].

In Spain, there are over 700,000 people affected by AD among a population of 49 million (of which 9.62 million are aged over 65 years) [6], with 40,000 new cases diagnosed annually [7]. However, reliable records on the epidemiology of AD stages are lacking. AD is estimated to be underdiagnosed in 50%-70% of cases and is often diagnosed at relatively advanced stages [1], with loss of functional autonomy. Unfortunately, there is no universal access to core AD biomarkers or a standardized neuropsychological assessment able to diagnose AD at the MCI stage, which is optimal according to clinical recommendations [8].

Early diagnosis of AD during initial symptomatic stages is crucial for patients, families, caregivers, and healthcare professionals. This allows for effective planning of patient care, appropriate referral to healthcare services, access to new therapeutic options, and involvement of patients in decision-making processes, including potential inclusion in clinical trials [1, 9, 10]. Currently, early symptoms, such as mild memory loss, are often misattributed to normal aging, delaying accurate diagnosis [1]. Addressing this misconception could heighten awareness of the development of diseases such as AD. Another key aspect is improving the diagnostic process, starting with brief cognitive screening tests, which currently show suboptimal performance in detecting MCI, particularly in younger individuals and/or those with higher levels of education [11]. Current diagnostic methods involve a comprehensive clinical evaluation, including brief cognitive tests and a neurological examination, followed by blood tests to rule out treatable causes of cognitive decline [12]. Additional assessments may include neuropsychological evaluation, structural and functional brain imaging, and cerebrospinal fluid (CSF) core biomarker analysis to support diagnosis [12]. There is inequitable access to diagnostic workup due to its ongoing advancements, the lack of universal access to approved diagnostic resources, limitations in healthcare infrastructure, cost, and the invasive nature of certain tests [13]. In addition, according to the updated National Institute on Aging-Alzheimer’s Association (NIA-AA) research framework, AD is defined biologically through the AT(N) system, which classifies biomarkers into three categories: amyloid (A), tau (T), and neurodegeneration (N) [14]. This framework provides a standardized approach for the diagnosis of MCI due to AD and improves diagnostic accuracy, a critical aspect of the diagnostic workup for AD. Diagnosing AD without AT(N) biomarkers reduces diagnostic reliability and hinders a comprehensive, patient-centered therapeutic approach [15, 16].

Most of the available therapies for AD alleviate the symptoms but do not address disease progression; however, new disease-modifying therapies (DMTs) have been shown to slow disease progression in early symptomatic stages [3, 5, 17]. This underscores the importance of earlier and accurate core biomarker-based diagnosis at initial symptomatic stages, such as MCI or mild dementia.

The cost of AD management significantly impacts overall healthcare costs. In southern Europe, the average annual cost per patient with dementia is €35,866, varying by disease stage and increasing with symptomatic progression [18]. Notably, 85% of the AD-related economic burden is covered by the patient and their family [1], even in countries such as Spain, where there is a universal healthcare system. This is associated with considerable emotional and physical strain, mainly anxiety and depression, faced by caregivers and direct family members [19-21].

Despite the substantial public health burden of AD in Spain, significant gaps remain in understanding the current epidemiological landscape, diagnostic practices, and the humanistic and economic impacts of the disease. Addressing these gaps is crucial to fully understanding the true impact of AD. Given the high prevalence and burden of AD among the elderly, more population-based data are essential to develop effective resource allocation and efficient healthcare management strategies. This study reviews recent literature (2019-2024) to capture the latest advancements in AD research, particularly in relation to new DMTs and updates to diagnostic criteria, aiming to support informed decision-making in policy and clinical practice.

## Review

Methods

A systematic literature review (SLR) was conducted to evaluate 1) epidemiology, 2) diagnostic workup, 3) patient journey, 4) humanistic burden (functionality and cognitive impairment in patients with AD, as well as health-related quality of life (HRQoL) of their caregivers), and 5) economic burden (resource utilization and direct and indirect costs) of AD in Spain. The recommendations included in the Preferred Reporting Items for Systematic Reviews and Meta-Analyses (PRISMA) were followed [22]. This SRL followed a standardized and unpublished protocol.

Data Sources and Search Strategy

The international databases PubMed/Medical Literature Analysis and Retrieval System Online (MEDLINE), Medicina en Español (MEDES), and Índice Bibliográfico Español en Ciencias de la Salud (IBECS) were searched using standardized search filters to identify relevant publications. Because this review aimed to characterize the epidemiological, diagnostic, humanistic, and economic burden of AD specifically within the Spanish healthcare context, databases with extensive coverage of Spanish biomedical literature were prioritized. In particular, MEDES and IBECS were included to capture studies published in Spanish-language journals that may be underrepresented in broader international databases.

Both Medical Subject Headings (MeSH) and free-text terms were used and combined with the Boolean connectors OR and AND to search the databases. The MeSH and free-text terms used are detailed in Table 1.

**Table 1:** Search strategy for PubMed/Medical Literature Analysis and Retrieval System Online (MEDLINE), Medicina en Español (MEDES), and Índice Bibliográfico Español en Ciencias de la Salud (IBECS)

Search strategy for PubMed/MedLine				
Disease				
1.	Alzheimer Disease [MeSH]			
2.	Alzheimer [tiab]			
Country				
3.	Spain [tw]			
4.	Spanish [tw]			
5.	Andalucia [tw]			
6.	Andalusía [tw]			
7.	Aragon [tw]			
8.	Asturias [tw]			
9.	Balear* [tw]			
10.	Canar* [tw]			
11.	Cantabria [tw]			
12.	Castilla [tw]			
13.	Cataluña [tw]			
14.	Catalunya [tw]			
15.	Catalonia [tw]			
16.	Ceuta [tw]			
17.	Valenciana [tw]			
18.	Valencian [tw]			
19.	Extremadura [tw]			
20.	Galicia [tw]			
21.	Madrid [tw]			
22.	Murcia [tw]			
23.	Melilla [tw]			
24.	Navarr* [tw]			
25.	Vasco [tw]			
26.	Euskadi [tw]			
27.	Basque [tw]			
28.	Rioja [tw]			
Clinical Burden				
29.	Epidemiologic factor [MeSH]			
30.	Incidence [MeSH]			
31.	Prevalence [MeSH]			
32.	Age of Onset [MeSH]			
33.	Mortality [MeSH]			
34.	Epidemiologic factors [tiab]			
35.	Incidence [tiab]			
36.	Prevalence [tiab]			
37.	Age of Onset [tiab]			
38.	Mortality [tiab]			
Diagnosis				
39.	Diagnosis [MeSH]			
40.	Risk factors [MeSH]			
41.	Cognitive Dysfunction [MeSH]			
42.	Dementia [MeSH]			
43.	Biomarkers [MeSH]			
44.	Diagnos* [tiab]			
45.	Diagnostic technique* [tiab]			
46.	Cognitive dysfunction [tiab]			
47.	Cognitive impairment [tiab]			
48.	Dementia [tiab]			
49.	Risk factors [tiab]			
50.	Biomarker [tiab]			
51.	Subjective cognitive complaint [tiab]			
52.	Self-reported memory complaint [tiab]			
Humanistic burden				
53.	Quality of life [MeSH]			
54.	Patient-Reported Outcome Measures [MeSH]			
55.	Quality of life [tiab]			
56.	Patient-Reported Outcome Measures [tiab]			
57.	Health-related quality of life [tiab]			
58.	Health status [tiab]			
Economic burden				
59.	Global Burden of Disease [MeSH]			
60.	Cost of Illness [MeSH]			
61.	Cost of Illness [tiab]			
62.	Costs and cost analysis [MeSH]			
63.	Economic burden [tiab]			
64.	Burden [tiab]			
65.	Cost [tiab]			
66.	Absenteeism [tiab]			
67.	Presenteeism [tiab]			
68.	Productivity [tiab]			
69.	Activity impairment [tiab]			
70.	Disability [tiab]			
71.	Economics [tiab]			
Disease management				
72.	Standard of care [MeSH]			
73.	Treatment patterns [tiab]			
74.	Standard of care [tiab]			
75.	Patient care management [MeSH]			
76.	Disease management [tiab]			
77.	Patient journey [tiab]			
((OR 1-2) AND (OR 3-28)) AND (OR 29-77)				
Search strategy for Medicina en Español (MEDES)				
Disease				
1.		Alzheimer [título/resumen/palabras_clave]		
Country				
2.		España [Todos los campos]		
3.		Andalucia [Todos los campos]		
4.		Aragon [Todos los campos]		
5.		Asturias [Todos los campos]		
6.		Balear* [Todos los campos]		
7.		Canar* [Todos los campos]		
8.		Cantabria [Todos los campos]		
9.		Castilla [Todos los campos]		
10.		Cataluña [Todos los campos]		
11.		Ceuta [Todos los campos]		
12.		Valenciana [Todos los campos]		
13.		Extremadura [Todos los campos]		
14.		Galicia [Todos los campos]		
15.		Madrid [Todos los campos]		
16.		Murcia [Todos los campos]		
17.		Melilla [Todos los campos]		
18.		Navarra [Todos los campos]		
19.		Vasco [Todos los campos]		
20.		Euskadi [Todos los campos]		
21.		Rioja [Todos los campos]		
Clinical Burden				
22.		Epidemiologi* [título/resumen/palabras_clave]		
23.		Incidencia [título/resumen/palabras_clave]		
24.		Prevalencia [título/resumen/palabras_clave]		
25.		Edad de inicio [título/resumen/palabras_clave]		
26.		Mortalidad [título/resumen/palabras_clave]		
Diagnosis				
27.		Diagnóstico [título/resumen/palabras_clave]		
28.		Factores de riesgo [título/resumen/palabras_clave]		
29.		Deterioro cognitivo[título/resumen/palabras_clave]		
30.		Demencia [título/resumen/palabras_clave]		
31.		Biomarcadores [título/resumen/palabras_clave]		
32.		Queja subjetiva de memoria [título/resumen/palabras_clave]		
33.		Pérdida subjetiva de memoria [título/resumen/palabras_clave]		
Humanistic burden				
34.		Calidad de vida [título/resumen/palabras_clave]		
35.		Resultados percibidos por el paciente [título/resumen/palabras_clave]		
Economic burden				
36.		Carga de la enfermedad [título/resumen/palabras_clave]		
37.		Coste de la enfermedad [título/resumen/palabras_clave]		
38.		Carga económica[título/resumen/palabras_clave]		
39.		Absentismo [título/resumen/palabras_clave]		
40.		Presencialismo [título/resumen/palabras_clave]		
41.		Productividad [título/resumen/palabras_clave]		
42.		Discapacidad [título/resumen/palabras_clave]		
43.		Económico [título/resumen/palabras_clave]		
44.		Economía [título/resumen/palabras_clave]		
Disease management				
45.		Calidad asistencial [título/resumen/palabras_clave]		
46.		Manejo [título/resumen/palabras_clave]		
47.		Estándar de calidad [título/resumen/palabras_clave]		
48.		Viaje del paciente [título/resumen/palabras_clave]		
49.		Flujo asistencial [título/resumen/palabras_clave]		
50.		Proceso asistencial [título/resumen/palabras_clave]		
51.		Indicador de calidad [título/resumen/palabras_clave]		
(1 AND (OR 2-21)) AND (OR 22-51)				
Search strategy for Índice Bibliográfico Español en Ciencias de la Salud (IBECS)				
Disease				
1.			Alzheimer [palabras del resumen]	
Country				
2.			España [Palabras]	
3.			Andalucia [Palabras]	
4.			Aragon [Palabras]	
5.			Asturias [Palabras]	
6.			Balear* [Palabras]	
7.			Canar* [Palabras]	
8.			Cantabria [Palabras]	
9.			Castilla [Palabras]	
10.			Cataluña [Palabras]	
11.			Ceuta [Palabras]	
12.			Valenciana [Palabras]	
13.			Extremadura [Palabras]	
14.			Galicia [Palabras]	
15.			Madrid [Palabras]	
16.			Murcia [Palabras]	
17.			Melilla [Palabras]	
18.			Navarra [Palabras]	
19.			Vasco [Palabras]	
20.			Euskadi [Palabras]	
21.			Rioja [Palabras]	
Clinical Burden				
22.			Epidemiologia* [Palabras del resumen]	
23.			Incidencia [Palabras del resumen]	
24.			Prevalencia [Palabras del resumen]	
25.			Edad de inicio [Palabras del resumen]	
26.			Mortalidad [Palabras del resumen]	
Diagnosis				
27.			Diagnóstico [Palabras del resumen]	
28.			Factores de riesgo [Palabras del resumen]	
29.			Deterioro cognitivo [Palabras del resumen]	
30.			Demencia [Palabras del resumen]	
31.			Biomarcadores [Palabras del resumen]	
32.			Queja subjetiva de memoria [Palabras del resumen]	
33.			Pérdida subjetiva de memoria [Palabras del resumen]	
Humanistic burden				
34.			Calidad de vida [Palabras del resumen]	
35.			Resultados percibidos por el paciente [Palabras del resumen]	
Economic burden				
36.			Carga de la enfermedad [Palabras del resumen]	
37.			Coste de la enfermedad [Palabras del resumen]	
38.			Carga económica [Palabras del resumen]	
39.			Absentismo [Palabras del resumen]	
40.			Presencialismo [Palabras del resumen]	
41.			Productividad [Palabras del resumen]	
42.			Discapacidad [Palabras del resumen]	
43.			Económico [Palabras del resumen]	
44.			Economía [Palabras del resumen]	
Disease management				
45.			Calidad asistencial [Palabras del resumen]	
46.			Manejo [Palabras del resumen]	
47.			Estándar de calidad [Palabras del resumen]	
48.			Viaje del paciente [Palabras del resumen]	
49.			Flujo asistencial [Palabras del resumen]	
50.			Proceso asistencial [Palabras del resumen]	
51.			Indicador de calidad [Palabras del resumen]	
(1 AND (OR 2-21)) AND (OR 22-51)				

Grey literature was reviewed as part of the search process. However, these findings referred to dementia in general and did not specify AD as the underlying etiology; therefore, they were excluded from the review.

Eligibility Criteria

The search included only cohort, case-control, and cross-sectional observational studies published from January 2019 to January 2024, in Spanish or English, involving a Spanish population of adults with AD diagnosis, MCI, or mild dementia due to AD (Table 2).

**Table 2:** Eligibility criteria HRQoL: health-related quality of life; AD: Alzheimer's disease; NA: not applicable

Study Characteristics	Eligible	Ineligible
Patient population	At least one of the following is required to be eligible: Adults with an AD diagnosis, Adults with mild cognitive impairment due to AD, Adults with mild dementia due to AD, Adults with subjective memory complaints related to AD	Non-Alzheimer’s dementias
Intervention - Treatment	NA	NA
Intervention - Comparison	NA	NA
Outcomes–	Epidemiology: Incidence, prevalence, mortality; Population characteristics: age, sex, risk factors, disease severity, age of onset; Clinical management: diagnostic techniques, biomarkers, patient journey, HRQoL of patients and caregivers; Resource utilization and costs: resource use, direct cost, pharmacological cost, productivity of patients and caregivers, absenteeism/presenteeism of patients and caregivers	NA
Study design	Observational studies (including cost studies)	Narrative reviews, Systematic reviews, and meta-analysis, Clinical trials, Opinion articles, Editorial, Pharmacoeconomic evaluations
Country	Spanish studies, Multi-country studies with the Spanish population (if disaggregated data are available)	Non-Spanish studies, Multi-country studies without Spanish population, Multi-country studies with no disaggregated data
Time Frame	Articles published: January 2019 to January 2024	Publications prior to 2019
Language	English, Spanish	Non-English, Non-Spanish

The search excluded narrative reviews, systematic reviews, meta-analyses, conference abstracts, clinical trials, opinion articles, editorials, pharmacoeconomic evaluations, studies including adults with a non-Alzheimer's dementia diagnosis, and studies without a Spanish population.

Study Selection

Two independent researchers selected eligible publications. Firstly, the investigators screened by title and abstract, discarding publications outside the scope of the review. Then, the full text of the selected publications was reviewed to ascertain the final eligibility for this SRL. Discrepancies were resolved by consensus or by involving a third team member. Only full-text articles were included.

Data Extraction and Quality Assessment

Data extracted included the author, the publication year, the objective of the study, the type of study, characteristics of the study population, epidemiology, diagnosis, patient journey, HRQoL of patients and caregivers, resource utilization, and costs. No formal statistical analysis was performed. The methodological quality and risk of bias of the included studies were assessed using the Newcastle-Ottawa Scale (NOS) [23]. This instrument was selected because the included studies were non-interventional observational designs, for which the NOS provides the most appropriate framework to evaluate both methodological quality and potential sources of bias. Two independent reviewers extracted the data and assessed the quality of the studies, and the discrepancies were solved by consensus.

Ethical Approval

This article is based on published studies and, therefore, does not involve studies with human participants or animals performed by the authors. Accordingly, as a systematic review of published literature, this study did not require evaluation by an ethics committee.

Results

**Figure 1:**
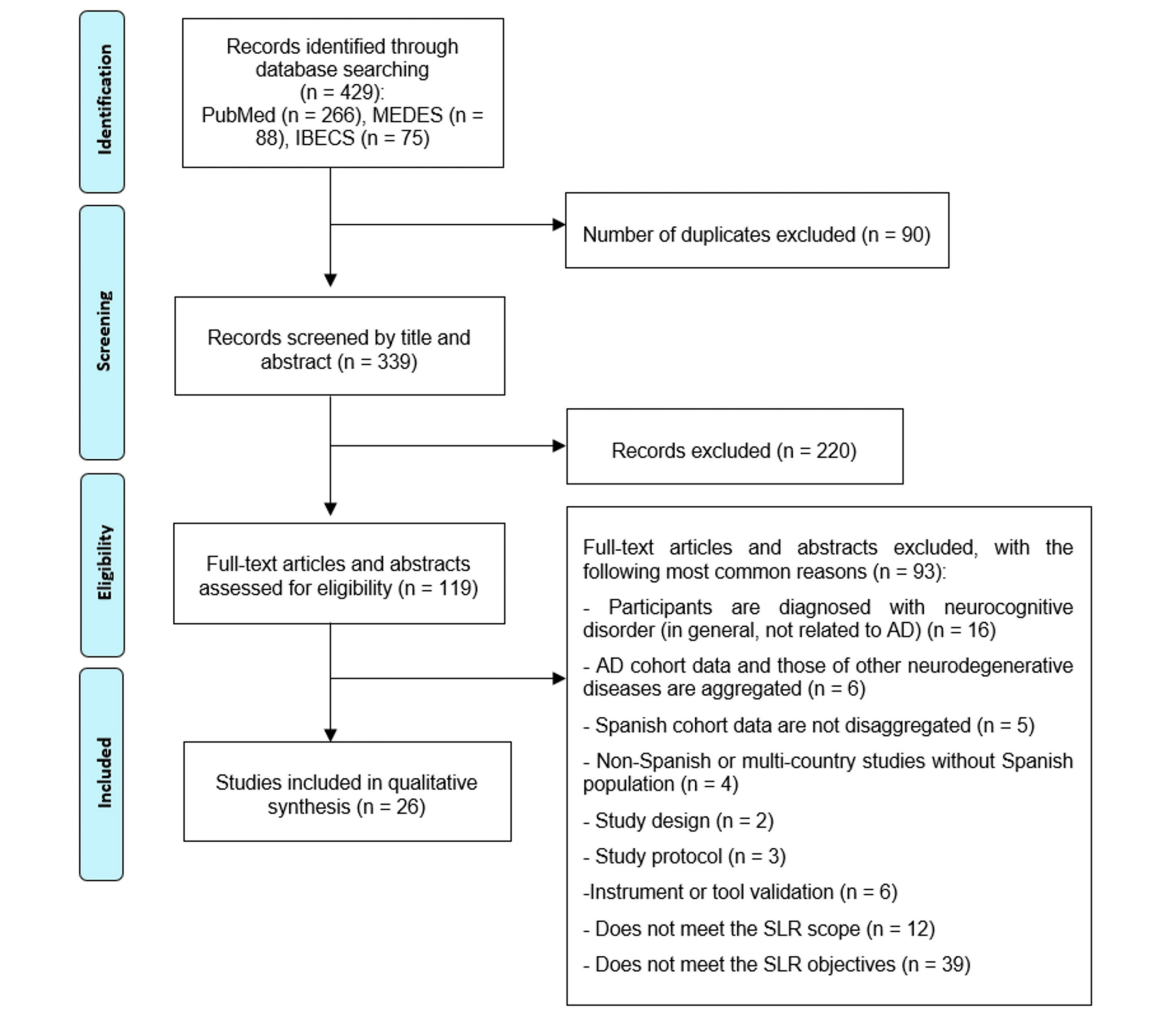
PRISMA flow diagram PRISMA: Preferred Reporting Items for Systematic Reviews and Meta-Analyses; MEDES: Medicina en Español; IBECS: Índice Bibliográfico Español en Ciencias de la Salud; AD: Alzheimer’s disease; SLR: systematic literature review

Out of the 429 articles initially identified, 26 publications were finally included in the SLR (Figure 1). The NOS quality assessment indicated a medium-to-high quality rating in most of the selected publications (score ≥5 out of a maximum of 9). Lower scores observed in some studies reflected the inclusion of descriptive cross-sectional or other non-comparative designs that did not fully align with the NOS framework, originally developed for cohort and case-control studies (Table 3). The characteristics of each publication are described in Table 4. Most of the publications included combined information about several variables of interest, such as epidemiology, diagnostic workup, patient journey, information about the caregivers and their HRQoL, resource utilization, and costs.

**Table 3:** Newcastle–Ottawa scale (NOS) assessment of methodological quality and risk of bias of included studies NA: Not aplicable/Not available; * descriptive cross-sectional studies or other types of studies (not cohort or case control studies) were analyzed according to the NOS method most similar to the specific study. #: “Item” refers to each criterion evaluated within the NOS domains (Selection, Comparability, and Outcome/Exposure). The presence of 0 or 1 indicates that the study fulfilled the criterion and was awarded one point.

Study	Study design analysis	Item								Total score
		1	2	3	4	5	6	7	8	
Andreu-Reinón (2020) [24]	Cohort	1	NA	1	1	1	1	1	1	7
Cámara-Calmaestra (2023) [25]*	Case control	1	0	1	1	1	1	1	NA	6
Cantón-Habas (2020) [26]	Case control	1	1	1	1	1	1	1	0	7
Casal Rodríguez (2019) [19]*	Cohort	0	0	1	0	NA	1	1	1	4
Darbà (2021) [27]	Cohort	1	0	1	0	NA	1	1	1	5
Durán-Gómez (2020) [20]*	Cohort	0	0	1	1	NA	1	1	1	5
Fernández Rubio (2023) [28]*	Cohort	1	0	1	0	NA	1	1	1	5
García-Escobar (2023) [29]*	Case control	1	0	0	1	2	1	1	1	7
Garcia-Ribas (2020) [30]*	Case control	1	0	1	1	1	1	1	0	6
Gomez-Gallego (2021) [31]*	Case control	1	1	0	0	1	1	0	0	4
Gómez Maldonado (2023) [32]*	Cohort	0	0	1	1	NA	1	1	1	5
Hernández-Padilla (2021) [33]*	Cohort	0	0	1	0	NA	1	1	1	4
Khandker (2020) [34]*	Cohort	1	0	1	0	NA	1	1	1	5
Kishita (2023) [35]*	Cohort	1	0	1	0	NA	1	1	1	5
Luque-Carrillo (2020) [36]*	Cohort	0	0	1	0	NA	1	0	1	3
Macedo (2021) [37]	Cohort	1	1	1	0	2	1	1	1	8
Mariezcurrena (2020) [38]*	Cohort	1	0	1	0	NA	1	1	0	4
Martínez Arrechea (2021) [39]*	Case control	1	1	0	1	1	1	1	1	7
Ponjoan (2020) [40]	Cohort	1	1	1	0	2	1	1	1	8
Poudevida (2022) [41]	Case control	1	1	1	1	2	1	1	0	8
Puig-Pijoan (2022) [42]	Cohort	1	1	1	0	2	1	1	1	8
Rosende-Roca (2022) [21]*	Cohort	0	0	1	0	NA	1	1	1	4
Roth (2023) [43]*	Cohort	1	0	1	0	NA	0	1	1	4
Ruiz-Fernández (2019) [44]*	Cohort	0	0	1	0	NA	1	1	1	4
Tortajada-Soler (2020) [45]*	Case control	1	0	1	1	1	1	1	1	7
Turró-Garriga (2020) [46]*	Cohort	0	0	1	0	NA	1	1	1	4

**Table 4:** Main characteristics of the selected studies * Descriptive cross-sectional studies for which no specific New Castle Ottawa Scale (NOS) evaluation method exist that were analyzed using the NOS methodology, classifying them as either case-control or cohort studies, depending on which study design they most closely resembled. Cross-sectional studies were considered retrospective if data were obtained from prior records, and prospective if data were collected during the study. AD: Alzheimer’s disease; aMCI: amnestic mild cognitive impairment; BDU® : Unified database (Base de datos unificados in Spanish); CU: cognitively unimpairedt; EPIC: European Prospective Investigation into Cancer and Nutrition cohort; HRQoL: Health-related quality of life; MCI: mild cognitive impairment; NA: not available; PCP: primary care physician; SIDIAP database: Information System for Research in Primary Care.

Author (year)	Geographical area	Recruitment year	Follow-up time	Type of study and study design	Population	Population size	Data source	Healthcare setting	Recovered variables
Andreu-Reinón (2020) [24]	National	1992–1996	20 years	Cohort study: longitudinal, prospective	General population (outpatient and hospital records)	774	EPIC cohort	Primary care, hospital	Demographic characteristics, epidemiology
Cámara-Calmaestra (2023) [25]	Regional (Andalusia)	2021–2022	NA	Case-control* study: Cross-sectional prospective	Geriatric population (nursing homes and living at home)	Total: 194 ≥ 65 yr AD patients: 114 ≥ 65 yr non-AD: 80	Study Survey	Nursing homes	Demographic characteristics, clinical management, resource utilization and costs, patient journey
Cantón-Habas (2020) [26]	National	2018–2019	NA	Case control study: cross-sectional, retrospective	Geriatric population (nursing homes and dementia-specific facilities)	221	Clinical history	Nursing homes and dementia-specific facilities	Demographic characteristics, epidemiology, clinical management, and HRQoL of patients
Casal Rodríguez (2019) [19]	Regional (Galicia)	2007–2008	NA	Cohort* study: cross-sectional, retrospective	Caregivers of patients with AD	175	Survey	Patient's home	Caregiver burden
Darbà (2021) [27]	National	2011–2016	NA	Cohort study: longitudinal, retrospective	General population (hospital records)	83	Spanish National Hospital Discharge Database	Hospital	Demographic characteristics, epidemiology, resource utilization, and costs
Durán-Gómez (2020) [20]	Regional (Extremadura)	2018–2019	NA	Cohort* study: cross-sectional, retrospective	General population (outpatient; patients and caregivers)	120	Survey	Patient's home	HRQoL of patients and caregivers
Fernández Rubio (2023) [28]	Regional (Andalusia)	2021	NA	Cohort* study: cross-sectional, retrospective	General population (on active therapies)	Total: 408,788, Patients with AD: NA	BDU^®^ study database	Health management area	Demographic characteristics, resource utilization, and costs
García-Escobar (2023) [29]	Regional (Catalonia)	NA	NA	Case-control* study: cross-sectional, prospective	General population (hospital records)	Total: 112 CU: 42 MCI: 35 Mild AD: 35	CORCOBIA study database	Hospital	Demographic characteristics Clinical management
Garcia-Ribas (2020) [30]	National	2019–2020	NA	Case-control* study: cross-sectional, Prospective	General population (caregivers and non-caregivers)	Total: 447; AD caregivers: 42; Non- Non-caregivers: 405	Survey	Same dwelling caregiver home, other relatives' home, nursing home	Demographic characteristics, patient journey, and caregivers
Gomez-Gallego (2021) [31]	NA	NA	NA	Case-control* study: cross-sectional, Prospective	General population (outpatient)	196	Clinical data and validated questionnaires	Primary health centers and dementia day centers	Demographic characteristics, clinical management, HRQoL of patients and caregivers
Gómez Maldonado (2023) [32]	National	2021–2022	NA	Cohort* study: cross-sectional, retrospective	Caregivers of patients with AD	171	Survey	Same dwelling, own home, nursing home	Demographic characteristics, resource utilization and costs, patient journey, and caregivers
Hernández-Padilla (2021) [33]	Regional (Andalusia)	2015–2015	NA	Cohort* study: cross-sectional, retrospective	Caregivers of patients with AD	255	Survey	Health management area	Demographic characteristics of caregivers
Khandker (2020) [34]	National	2015–2016	NA	Cohort* study: cross-sectional, retrospective	General population, including patients with cognitive impairment	Total: 846; MCI: 48; Mild AD: 334; moderate AD: 386; severe AD: 78	Survey	Primary care, hospitalization, and institutionalized	Demographic characteristics, epidemiology, resource utilization, and costs
Kishita (2023) [35]	National	NA	NA	Cohort* study: longitudinal, retrospective	Caregivers (family) of patients with AD	Caregivers: 322; patients: 322	Clinical history	NA	Demographic characteristics, epidemiology
Luque-Carrillo (2020) [36]	Regional (Andalusia)	NA	NA	Cohort* study: cross-sectional, retrospective	Patients with AD and their caregivers	69	Survey	Day care center	Demographic characteristics, HRQoL of patients and caregivers
Macedo (2021) [37]	Regional (Castile and Leon)	NA	NA	Cohort study: cross-sectional, prospective	Geriatric population	111	Clinical history	NA	Demographic characteristics, epidemiology, and clinical management
Mariezcurrena (2020) [38]	Regional (Navarra)	2019–2019	NA	Cohort* study: cross-sectional, prospective	Caregivers and families of patients with patients	76	Survey, clinical data, and validated questionnaires	NA	Demographic characteristics of caregivers
Martínez Arrechea (2021) [39]	Regional (Andalusia)	Phase 1: 2017; Phase 2: 2019–2019	NA	Case-control* study: cross-sectional, Retrospective	Geriatric population (institutionalized)	Total: 178; AD: 114 (64%)	Clinical history	Nursing homes	Resource utilization and costs
Ponjoan (2020) [40]	Regional (Catalonia)	2016–2016	NA	Cohort study: cross-sectional, retrospective	General population (>65 yr)	Total: 1,048,956; AD: 39,448; other: 1,009,508	SIDIAP database	Primary care	Demographic characteristics, epidemiology, and clinical management
Poudevida (2022) [41]	National	2013–2014	NA	Case-control study: longitudinal, prospective	Caregivers of patients with AD	221	Clinical data and validated questionnaires	Hospital and outpatient	Demographic characteristics, clinical management, and caregivers
Puig-Pijoan (2022) [42]	Regional (Catalonia)	2014–2019	19 months	Cohort study: cross-sectional, prospective	Patients with AD and unimpaired individuals (60-85 yr)	Total: 125; CU: 42; aMCI: 35; mild AD: 48	Clinical data and validated questionnaires	Hospital and outpatient	Demographic characteristics, clinical management
Rosende-Roca (2022) [21]	Regional (Catalonia)	2011–2020	NA	Cohort study: cross-sectional, prospective	Patients with AD and their caregivers	1065	Ace Alzheimer Barcelona center database	Alzheimer Care Center	Demographic characteristics, clinical management, patient journey, and caregivers
Roth (2023) [43]	National	2021–2021	NA	Cohort* study: cross-sectional, prospective	Healthcare providers	241	Survey	Healthcare System	Epidemiology, clinical management. resource utilization and costs, patient journey
Ruiz-Fernández (2019) [44]	Regional (Andalusia)	2015–2015	NA	Cohort* study: cross-sectional, prospective	General population (healthcare records)	255	Case manager nurses’ caregiver database	Health Management Area	Demographic characteristics, HRQoL of patients and caregivers
Tortajada-Soler (2020) [45]	Regional (Castile and Leon)	2019	NA	Case-control* study: cross-sectional, retrospective	Patients with AD and controls (>65)	Total: 200; AD: 100; Non-AD: 100	Clinical history	Alzheimer Care Center	Demographic characteristics, resource utilization, and costs
Turró-Garriga (2020) [46]	Regional (Catalonia)	2018–2019	24 months	Cohort* study: cross-sectional, prospective	Caregivers of patients with AD (clinical registries)	147	Survey	Healthcare System (memory clinics)	Demographic characteristics, clinical management, HRQoL of patients, resource utilization and costs, patient journey, and caregivers

Population Characteristics

Demographic characteristics: The studies included in this SLR were highly variable in terms of the contexts in which they were conducted (e.g., general population, nursing homes, daycare centers, hospital records), study design, and AD disease stage evaluated. However, in general, the AD population included in these studies had a higher proportion of women (60%-70%) than men [19-21, 24-27, 29, 31-37, 40, 42, 44, 46], and the mean age of patients with AD ranged from 70 to 82 years [19, 20, 26, 29, 31, 32, 35, 36, 42, 46]. Patients with AD were more likely to have low and middle socioeconomic status, while non-AD patients predominantly fell into middle status [19, 25]. Patients with AD generally had a lower education level (39.8-93.8% primary education or lower, 6.2-60.2% secondary or higher education) (Table 5) [21, 25].

**Table 5:** Main demographic characteristics of the study population on reporting publications Population age is represented as the mean (years). Disease severity is represented as N (%): Percentages are calculated out of the total number of patients with AD except in García-Escobar (2023) [29] and Puig-Pijoan (2022) ) [38], which included cognitively unimpaired (CU) individuals and population with mild-stage AD or amnestic mild cognitive impairment (aMCI), and percentages are 100% for each specific disease population or * are calculated considering the total number of participants in the study (CU+MCI+Mild AD). Sex and place of residence are represented as N (%). AD: Alzheimer’s disease; CU: cognitively unimpaired; MCI: mild cognitive impairment; NA: not applicable/not available; n.d: not defined; SD: standard deviation.

Author (year)	Sex (female, %)	Population age (years ± SD)	Population	Disease severity	Place of residence	Monthly income	Education level
Andreu-Reinón (2020) [24]	453 (58.5%)	Age at recruitment (N, %) <65: 751, 97.7% ≥ 65: 23, 2.3%	General population (outpatient and hospital records)	NA	NA	NA	NA
Cámara-Calmaestra (2023) [25]	Overall: 131 (67.5%), AD: 86 (75.4%), CU: 45 (56.3%)	Overall: 79.0 ± 8.6 AD: 82.9 ± 7.2 CU: 73.5 ± 7.2	Geriatric population (nursing homes and living at home)	NA	AD: 114 (100 %) nursing homes CU: 80 (100%) home	AD: Low 26 (22.8%), medium 77 (67.5%), high 11 (9.7%). CU: Low 5 (6.3%), medium 72 (90%), high 3 (3.8%)	AD: No studies: 42 (36.8%), Primary education: 65 (57.0%), Secondary education: 6 (5.3%), University: 1 (0.9%), CU: No studies: 5 (6.2%), Primary education: 635 (43.8%), Secondary education: 18 (22.5%), University: 22 (27.5%)
Cantón-Habas (2020) [26]	168 (76%)	79.1 ± 8.6	Geriatric population (nursing homes and dementia-specific facilities)	NA	Nursing homes: 107 (48.4%)	NA	NA
Casal Rodríguez (2019) [19]	AD: 136 (77.7%), Caregivers: 124 (70.9%)	AD: 78.3 ± 8.2 Caregivers: 58.1 ± 14.2	Caregivers of AD patients	Mild AD: 62 (35.8%) Moderate AD: 66 (37.7%) Severe AD: 47 (26.7%)	NA	AD: < 900€: 127 (75.6%) 900–2,400€: 40 (22.9%) > 2,400€: 4 (2.3%) n.d.: 4 (2.3%), Caregivers: < 900€: 95 (54.3%) 900–2,400€: 66 (37.7%) > 2,400€: 10 (5.7%) n.d.: 4 (2.3%)	AD: NA, Caregivers: No studies: 6 (3.4%), Primary education: 57 (32.6%), Secondary education: 67 (38.3%), University: 45 (25.7%)
Darbà (2021) [27]	(62.0%)	80.4 ± 8.3	General population (hospital records)	NA	NA	NA	NA
Durán-Gómez (2020) [20]	AD: 78 (65%), Caregivers: 104 (86.7%)	AD: 73.2 ± 5.6, Caregivers: 50.5 ± 4.2	General population (not institutionalized)	NA	NA	NA	AD: NA, Caregivers: No studies: 7 (5.8%) Primary education: 48 (40%), Secondary education: 42 (35%)m University: 23 (19.2%)
García-Escobar (2023) [29]	CU: 21 (50%), MCI: 19 (54.3%), Mild AD: 24 (68.6%)	CU: 71.3 ± 5.5, MCI: 74.5 ± 4.3, Mild AD: 74.3 ± 6.3	General population (Hospital records)	MCI: 35 (100%); 35 (*31.3) Mild AD: 35 (100%); 35 (*31.3) Moderate AD: NA Severe AD: NA	NA	NA	NA
Garcia-Ribas (2020) [30]	Caregivers: 30 (71.4%)	Caregivers: ≤30 years: 1 (2.9%) 31–40 years: 5 (11.9%) 41–50 years: 20 (47.6%) 51–60 years: 16 (38.1%) >60 years: 0 (0%)	General population (caregivers and non-caregivers)	AD: MCI: NA, Mild AD: 11 (26.2%), Moderate AD: 8 (19.1%), Severe AD: 23 (54.8%)	AD: Own home: 27 (64.3%), Caregiver home: 1 (2.4%), Other relatives home: 4 (9.5%), Nursing home: 10 (23.8%)	NA	AD: NA, Caregivers: Secondary education: 3 (7.1%), Vocational training: 3 (7.1%), University: 36 (85.8%)
Gomez-Gallego (2021) [31]	119 (60.7%)	Female: 78.1 ± 9.2	General population (not institutionalized)	NA	NA	NA	NA
Gómez Maldonado (2023) [32]	117 (68.8%)	79.1 ± 7.4	Caregivers of AD patients	MCI: NA Mild AD: 24 (14.0%) Moderate AD: 75 (43.9%) Severe AD: 72 (42.1%)	Same dwelling: 144 (84.2%), Own home: 19 (11.1%), Nursing home: 8 (4.7%)	NA	NA
Hernández-Padilla (2021) [33]	AD: 149 (58.4%), Caregivers: 218 (85.5%)	AD: Age at recruitment (N, %) ≤ 70 years: 29 (11.4%) 71–80 years: 114 (44.7%) ≥81 years: 112 (43.9%) Caregivers: 55.35 ± 12.35	Caregivers of patients with AD	NA	NA	NA	AD: NA, Caregivers: No studies: 46 (16.5%), Primary studies: 107 (42%), Secondary studies: 71 (27.8%), University: 36 (13.7%)
Khandker (2020) [34]	Overall: 3291 (53.7%), Very mild AD: 312 (47.3%), Mild AD: 1308 (53.0%), Moderate AD: 1426 (54.8%), Severe AD: 245 (60.0%)	Overall: 76.3 ± 8.8, Very mild AD: 71.8 ± 9.4, Mild AD: 75.1 ± 8.7, Moderate AD: 78.1 ± 8.1, Severe AD: 80.2 ± 8.6	General population, including patients with cognitive impairment	MCI: NA, Mild AD: 334 (41.9%), Moderate AD: 386 (48.4%), Severe AD: 78 (9.8%)	Institutionalized Mild AD: 8 (2.4%), Moderate AD: 57 (14.8%), Severe AD: 22 (28.6%)	NA	NA
Kishita (2023) [35]	AD: 209 (64.9%), Caregivers: 223 (69.3%)	AD: 79.9 ± 8.9, Caregivers: 62.28 ± 12.77	Caregivers (family) of patients with AD	NA	NA	NA	NA
Luque-Carrillo (2020) [36]	AD: 48 (69.6%), Caregivers: 49 (71%)	AD: 79. 8 ± 7.9, Caregivers: 58.77 ± 11.23	Patients with AD and their caregivers	NA	NA	NA	AD: NA, Caregivers: No studies: 9 (13%), Primary studies: 15 (21.7%), Secondary studies: 14 (20.3%), University studies: 20 (29%), Incomplete studies (secondary or university studies): 11 (15.9%)
Macedo (2021) [37]	Overall: 74 (66.6%), CU: 12 (50%), MCI: 15 (68.2%), Mild AD: 12 (50%), Moderate AD: 18 (81.8%), Severe AD: 17 (89.5%)	NA	Geriatric population	MCI: 23 (20.7%), Mild AD: 24 (21.6%), Moderate AD: 22 (19.8%), Severe AD: 19 (17.1%)	NA	NA	NA
Ponjoan (2020) [40]	Overall: 599,942 (57.2%), AD: 28,360 (71.9%), Other: 571,582 (56.6%)	Overall: 75.9 ± 7.9, AD: 83.1 ± 6.6, Other: 75.6 ± 7.8	General population (>65 yr)	NA	NA	NA	NA
Puig-Pijoan (2022) [42]	CU: 21 (50%), aMCI: NA, Mild AD: 35 (72.9%)	CU: 71.3 ± 5.5, aMCI: NA, Mild AD: 73.7± 6.1	Patients with AD and unimpaired individuals (60-85 yr)	MCI: 35 (100%); 35 (*28%), Mild AD: 48 (100%); 35 (*38.4%) Moderate AD: NA, Severe AD: NA	NA	NA	NA
Rosende-Roca (2022) [21]	AD patients: 648 (60.8%), Caregivers: 670 (62.9%)	AD patients: 80.1 ± 7.9 Caregivers: 69.0 ± 12.0	Patients with AD and their caregivers	NA	NA	NA	AD: Primary or lower: 424 (39.8%), Secondary or higher: 641 (60.2%), Caregivers: Primary or lower: 654 (61.4%), Secondary or higher: 411 (38.6%)
Ruiz-Fernández (2019) [44]	AD: Early-stage: 60 (55.6%), Middle-stage: 89 (60.5%). Caregivers of patients with early-stage AD: 88 (81.5%), Caregivers of Middle-stage AD patients: 130 (88.4%)	AD: Early-stage patients: 76.6 ± 8.2, Middle-stage patients: 80.0 ± 6.3, Caregivers: NA	General population (healthcare records). Patients with AD and their caregivers	MCI: NA, Mild AD: 108 (42.4%), Moderate AD: 147 (57.6%), Severe AD: NA	NA	NA	AD: NA, Caregivers of patients with early-stage AD: No studies: 21.3 (23%), Primary studies: 42 (38.9%), Secondary studies: 30 (27.8%), University: 13 (12%), Caregivers of patients with middle-stage AD: No studies: 12.9 (19%), Primary studies: 65 (44.2%), Secondary studies: 41 (27.9%), University: 22 (15%)
Tortajada-Soler (2020) [45]	Overall: (61%), Non-AD: (53%), AD: (69%)	NA	Patients with AD and controls (>65)	NA	NA	NA	NA
Turró-Garriga (2020) [46]	AD: 92 (62.6%), Caregivers: 90 (61.2%)	AD: 78.4 ± 5.9, Caregivers: 65.0 ± 12.9	Caregivers of patients with patients (clinical registries)	NA	Own home: 91.2 %	Household income > 25,000€ per year: 43 (29.5%)	Caregivers: Primary school or lower: 81 (55.1%)

Disease severity: Disease severity, from MCI to dementia (mild, moderate, and severe), was described in eight publications (Table 5). The distribution of subpopulations varied between studies, with the moderate and severe forms of AD dementia being the most commonly represented (19.1%-57.6% moderate AD; 9.8-54.8% severe AD) [19, 29, 30, 32, 34, 37, 42, 44]. Only two studies included patients with MCI due to AD [29, 42]. One study showed that institutionalization of patients with AD increased as the severity of the disease progressed (2.4% for mild AD, 14.8% for moderate AD, and 28.6% for severe AD) (Table 5) [34].

Epidemiology

**Figure 2:**
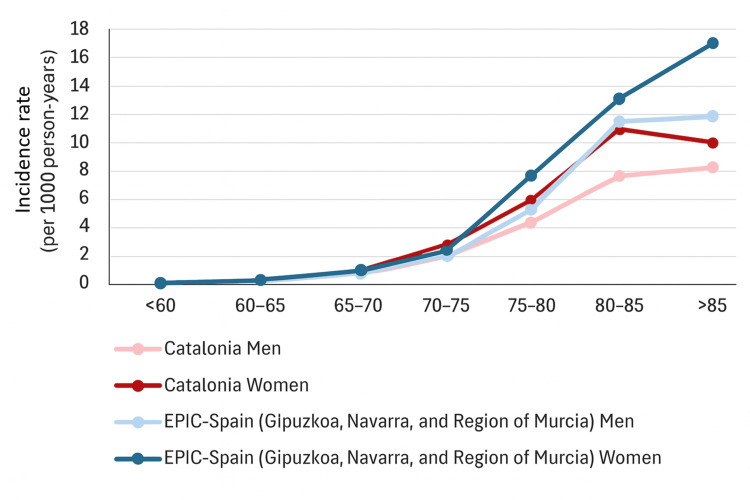
Incidence rates of AD per 1,000 person-years, stratified by age and sex Incidence of AD in Catalonia in 2016 (red; Ponjoan et al. [40]) and in EPIC-Spain Dementia Cohort between 1992 and 1996 (blue; Andreu-Reinón et al. [24]). AD: Alzheimer's disease; EPIC: European Prospective Investigation into Cancer and Nutrition

Incidence: Three publications reported data on the incidence of AD dementia, two of them at the national level (Spain) [24, 27], and one at a regional level (Catalonia) [40]. The European Prospective Investigation into Cancer and Nutrition (EPIC) Spain Dementia Cohort study included data from the general population aged 30-70 years, recruited between 1992 and 1996, with 20 years of follow-up, including in-hospital or primary care registries. The study specifically comprises data from centers in three provinces of Spain (Gipuzkoa and Navarra in the North and the Region of Murcia in the South), for which there was available information on the incidence of dementia (Figure 2) [24]. This study found that the incidence of AD dementia in people ≥65 was 2.8 per 1,000 inhabitants (5.5 per 1,000 inhabitants after age-standardized rate adjustment), 3.26 per 1,000 person-years (6.20 after age-standardized rate adjustment) in women, and 2.36 (4.59 after adjustment) in men [24]. Another study performed in 2016 in Catalonia with data from the general population primary care registry showed a slightly higher incidence of dementia among people ≥65, estimated as 4.2 per 1,000 inhabitants, 5.6 per 1,000 inhabitants (4.9 after age-sex standardization) in women, and 3.5 per 1,000 inhabitants (3.5 after age-sex standardization) in men [40]. A third study based on records of inpatient and outpatient admissions from a database of hospital discharges in Spain reported a mean national in-hospital incidence of AD dementia of 0.37 per 1,000 inhabitants (mean age: 80.4 years, SD: 8.1) between 2011 and 2016 [27]. This study also provided mean incidence rates by sex: 0.436 per 1,000 inhabitants in women (62.0% of the patients) and 0.294 per 1,000 inhabitants in men [27].

Prevalence: Four publications reported data on the prevalence of AD dementia in people aged ≥65 years (Table 6); one did so in the general population in a region of Spain, while the other three reported it in patients with dementia, with varying inclusion criteria [26, 35, 43]. Ponjoan and colleagues reported an estimated 3.8% (3.3% age-standardized and 3.6% sex-standardized) prevalence of AD dementia in the general population using the Information System for Research in Primary Care (SIDIAP) database [40]. The other three studies evaluated data collected from nursing homes [26] or reported by either healthcare providers [43] or caregivers of patients with dementia [35]; overall, the prevalence of AD dementia ranged from 33.5% to 55.6% [26, 35, 43]. Two articles reported differences by sex, with a higher prevalence in women (3.8%-35.7%) than men (2.4%-26.4%) [26, 40].

**Table 6:** Prevalence of AD dementia in the specific populations analyzed AD: Alzheimer's disease; HCP: health care professional; MCI, mild cognitive impairment; NA, not applicable. Data are presented as % (N). *AD% % is not specified.

Author (year)	Geographical area and study population	Setting	Inclusion criteria	Prevalence of AD dementia in the general population	Prevalence of AD dementia in the study population
Ponjoan (2020) [40]	Regional: 1,048,956 people registered in SIDIAP*	General population (≥65 years)	AD cases identified by diagnosis code or dementia drug use, excluding cases with other dementia types, recent cerebrovascular disease, or Parkinson’s in individuals aged ≥ 65	Overall: 3.8% (3.1% after age-sex-standardization) (N = 39,448), Women: 4.7% (3.8% after age-sex-standardization), Men: 2.5% (2.4% after age-sex-standardization)	NA
Cantón-Habas (2020) [26]	National: 221 125 people without dementia, 96 participants with dementia (of whom 74 subjects had AD)	Geriatric population (nursing homes and one dementia-specific facility)	People without dementia/AD: Person ≥ 65 years not presenting with a diagnosis of dementia or AD Dementia/AD: Person ≥ 65 years with dementia and/or AD and a score of 5–7 on the Global Deterioration Scale (GDS)	NA	33.5% (N = 74) Women: 35.7% Men: 26.4%
Roth (2023) [43]	National: 449 AD: 187 Vascular dementia: 107 Lewy body dementia: 35 Dementia linked to Parkinson’s disease: 34 Non-etiologic diagnoses of symptoms: 73 Other: 13	Survey of healthcare providers	Patients with dementia (most of them ≥66 years)	NA	41.6% (N = 187)
Kishita (2023) [35]	National: 322 (attended by caregivers), AD: 179, Other type of dementia: 92	Survey to caregivers (family) of patients with AD	Patients with dementia or related disorders ≥70 years attended by caregivers	NA	55.6 % (N = 179)

Mortality: Only one publication reported AD-associated mortality data [27]; specifically, the mean in-hospital AD-associated mortality rate was 9.5% (between 2011 and 2016, at the national level), mainly due to respiratory complications or heart failure [27].

Diagnosis

**Figure 3:**
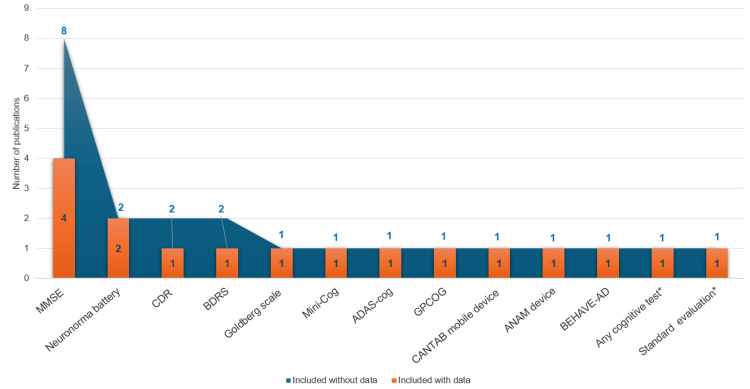
Number of publications reporting cognitive test use with (orange) and without score data (blue). * Not specified MMSE: Mini-Mental State Examination; CDR: clinical dementia rating; BDRS: Blessed Dementia Rating Scale; ADAS-cog: Alzheimer’s Disease Assessment Scale–Cognitive Subscale; GPCOG: General Practitioner Assessment of Cognition; CANTAB mobile device test: Cambridge Neuropsychological Test Automated Battery; ANAM: Automated Neuropsychological Assessment Metrics; BEHAVE-AD: Behavioural Pathology in Alzheimer’s Disease Rating Scale

**Figure 4:**
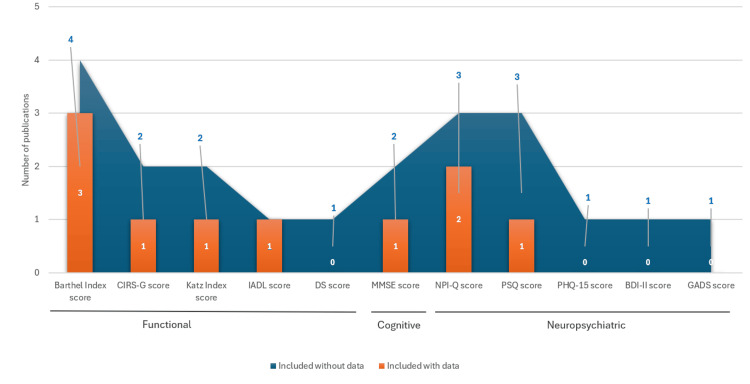
Number of publications reporting functionality and cognitive test use with (orange) and without score data (blue). AD: Alzheimer’s disease; BDI-II: Beck Depression Inventory; CIRS-G: Cumulative Illness Rating Scale–Geriatric; DS: Dependence scale; GADS: Goldberg Anxiety and Depression Scale; IADL: Katz’s Instrumental Activities of Daily Living Index; MMSE: Mini-Mental State Examination; NPI-Q: Neuropsychiatric Inventory Questionnaire; PHQ-15: Patient Health Questionnaire; PSQ: Psychosocial Support Questionnaire.

Clinical assessment instruments. Information about the cognitive tests used for diagnosing AD was reported in eleven publications, which evaluated people with AD and dementia, with data extracted from primary care and hospital registries (Figure 3). The variability in the data was attributed to the heterogeneity in study design. Most of the studied populations presented data of moderate AD dementia and mainly evaluated cognitive status with brief cognitive tests, the most common being the Mini-Mental State Examination (MMSE). This test is a standardized tool, commonly used to grade AD severity, that assesses global cognitive function (score range 0-30), with lower scores indicating greater cognitive impairment. The average MMSE score ranged from 13.9 to 19.4 [21, 29, 31, 36, 37, 40, 43, 46], suggesting that the mild AD population is underrepresented in these studies. The presence and severity of neuropsychiatric symptoms, very common in AD, were mainly evaluated with the Neuropsychiatric Inventory Questionnaire (NPI-Q) and the Psychosocial Support Questionnaire (PSQ); the studies using these questionnaires evaluated only people with mild-to-moderate AD dementia [31, 44, 46]. Several studies showed that the presence of neuropsychiatric symptoms and/or their severity increases with disease progression. Specifically, Ruiz-Fernández and colleagues reported a mean ± SD NPI-Q of 6.9 ± 2.3 and 8.0 ± 2.3 in patients with mild and moderate AD dementia, respectively [44]; Turró-Garriga reported a mean ± SD NPI-Q score of 4.0 ± 3.7 in patients with mild-to-moderate AD [46]; and Gómez-Gallego et al. showed a mean ± SD PSQ score of 16.0 ± 3.5 and 16.8 ± 3.2 in women and men, respectively, in patients with moderate AD [31]. Moreover, seven publications also evaluated the functional impairment associated with cognitive decline in people with AD dementia (Figure 4) using different tests such as the Barthel Index, which assesses the autonomy of patients with AD in activities of daily living [23, 26, 36, 44]. The percentage of patients with total dependency ranged between 40.6% and 61.5% and correlated with disease severity [26, 36]. According to the Barthel Index, only 1.4% of patients had low dependence [36], while 7.3%-49.4% had moderate dependency [26, 36, 44], and 31.3%-74.8% were highly dependent [26, 44]. The most frequently used tool to assess disease severity was the Global Deterioration Scale (GDS), with scores ranging from 4 to 7 [20, 26, 29, 41, 42, 46].

Medical exams for differential diagnosis: Two studies reported information about general diagnostic evaluation: one focused on patients with AD attending memory clinics, and the other consisted of a survey examining usual practices of physicians [43, 46]. The most frequently reported clinical assessments were anamnesis and neurological physical examinations (82.8%-89.1%), highlighting the importance of interviews as a first step in the diagnostic workup to evaluate cognitive, neuropsychiatric, and functional symptoms. Other examinations used to a lesser extent during AD diagnostic workup were computed tomography (57.5%-62.6%) or magnetic resonance imaging (MRI) (36.1%-59.4%), as well as laboratory tests for differential diagnosis and detection of treatable/modifiable causes of cognitive impairment (58.5%-71.3%) [43, 46].

AD biomarkers: Only one study reported the use of specific AD biomarkers (amyloid positron emission tomography (PET)) in clinical practice (28.3%), as reported by specialists participating in a survey [43]. This study also reported that 27.3% of specialists used CSF biomarkers in their clinical practice [43]. Other studies examined CSF biomarkers, based on the AT(N) framework, in a research context [29, 36].

Patient journey

Six publications reported data related to the patient journey [21, 25, 30, 32, 43, 46]. The time to final diagnosis of AD dementia from the onset of symptoms was described in two studies as under six months (26.2% of the patients), one to two years (38.1%), and more than two years (28.6%) [30, 32]. The diagnosis was carried out mainly by neurologists (78.6%) [25, 30], followed by geriatricians (9.5%) and psychiatrists (2.4%) [30, 43]. Moreover, the follow-up appointments for patients with AD attending memory clinics were conducted by primary care physicians (70.1%) and neurologists (91.8%), with 63.3% of the cases involving additional neuropsychological assessments [46]. One publication indicated that the first medical consultation for symptoms of cognitive impairment or cognitive complaints was made by the patients or their families (62.4%), followed by healthcare professionals (36.3%) (Table 7) [43]. As a result of the medical consultation, 43.9% of patients received a diagnosis, and 25.1% were referred to another specialist for further testing or diagnosis (Table 7) [43].

**Table 7:** Patient journey during AD in Spain NA: not applicable/not available; AD: Alzheimer's disease; PCP: primary care physician; Unless specified, data are presented as N (%).

Author (year)	Time to first reported symptoms to diagnosis	Specialty for diagnosis; N (%)	Specialty for follow-up
Cámara-Calmaestra (2023) [25]	NA	Neurologists	NA
Garcia-Ribas (2020) [30]	6 months–1 year: 11 (26.2%), 1–2 years: 16 (38.1%), ˃2 years: 12 (28.6%), Unknown: 3 (7.1%)	PCPs: 4 (9.5%), Neurologist: 33 (78.6%), Geriatrician: 4 (9.5%), Psychiatrist: 1 (2.4%)	NA
Gómez Maldonado (2023) [32]	Years (Mean ± SD) 1.8 ± 2.3	NA	NA
Roth (2023) [43]	NA	PCP: 53 (35.8%), Other specialist: 134 (44.5%)	NA
Turró-Garriga (2020) [46]	NA	NA	70.1% primary care, average 1.8 visits. 91.8% other specialist appointments: neurologist. Neuropsychological assessments were conducted in 63.3% of these visits.

Comorbidities: Nine publications described the presence of comorbidities in the AD population [19, 20, 24-27, 34, 40, 45]. Interestingly, the prevalence of comorbid conditions increased with the severity of the disease [34]. The mean number of concomitant conditions suffered by AD patients varied between 1.88 and 3.11 [19, 20]. The most frequent comorbidities were hypertension (32.5%-69.7%) [24, 40], heart diseases (including myocardial infarction: 0.5%-11.8% [24, 34], cardiac dysrhythmias: 13.89% [27], congestive heart failure: 3.07%-11.8% [27, 34, 40], and coronary artery disease: 10.1% [40]), other cardiovascular diseases (6.9%-61.4%) [25, 34, 45], and cerebrovascular disease (1%-16.9%) [24, 25, 40, 45]. Hyperlipidemia (25.0%-56.1%) [27, 35], diabetes mellitus (11.5-31.6%) [24, 25], and depression (8.6%-28.5%) [27, 40] were also reported. Table 8 includes two studies that examine comorbidities in populations with and without AD and one study that shows comorbidities according to AD severity; hyperlipidemia, hypertension, and heart disease are the most frequent comorbidities among patients with AD [25, 34, 45].

**Table 8:** Comorbidities Comorbidities presented as N (%) AD: Alzheimer’s disease, MCI: mild cognitive impairment, NA: not applicable/not available; TIAs: transient ischemic attacks

Author (year)	Disease severity	Cardiometabolic diseases						Others
		Diabetes mellitus	Hyperlipidemia	Hypertension	Cerebrovascular disease	Heart disease	Other cardiovascular diseases	Depression
Cámara-Calmaestra (2023) [25]	Total	46 (23.7%)	NA	NA	NA	NA	108 (55.7%)	28 (14.4%)
	AD	36 (31.6%)				NA	70 (61.4%)	21 (18.4%)
	Non-AD	10 (12.5%)				NA	38 (47.5%)	7 (8.8%)
Khandker (2020) [34]	Total	249 (29.7%)	NA	517 (61.8%)	Stroke 72 (8.6%), TIAs 52 (6.2%), Cerebrovascular disease: 76 (9.1%)	Myocardial infarction: 63 (7.5%), Congestive heart failure: 56 (6.7%)	Peripheral vascular disease: 71 (8.5%)	226 (27.0%)
	MCI	9 (18.8%)		20 (41.7%)	Stroke 5 (10.4%), TIAs 2 (4.2%), Cerebrovascular disease: 3 (6.3%)	Myocardial infarction: 3 (6.3%), Congestive heart failure: 2 (4.2%)	Peripheral vascular disease: 6 (12.5%)	11 (22.9%)
	Mild AD	89 (26.9%)		182 (55.0%)	Stroke 22 (6.6%), TIAs 16 (4.8%), Cerebrovascular disease: 22 (6.6%)	Myocardial infarction: 17 (5.1%), Congestive heart failure: 17 (5.1%)	Peripheral vascular disease: 23 (6.9%)	82 (24.8%)
	Moderate AD	123 (32.2%)		261 (68.3%)	Stroke 37 (9.7%), TIAs 29 (7.6%), Cerebrovascular disease 43 (11.3%)	Myocardial infarction: 34 (8.9%), Congestive heart failure: 28 (7.3%)	Peripheral vascular disease: 32 (8.4%)	107 (28.0%)
	Severe AD	28 (36.8%)		54 (71.1%)	Stroke 8 (10.5%), TIAs 5 (6.6%), Cerebrovascular disease: 8 (10.5%)	Myocardial infarction: 9 (11.8%), Congestive heart failure: 9 (11.8%)	Peripheral vascular disease: 10 (13.2%)	26 (34.2%)
Tortajada-Soler (2020) [45]	AD group	19%	45%	NA	1%	31%	Peripheral vascular disease: 21%, Atrial fibrillation: 7%	27%
	Non-AD	20%	39%		5%	26%	Peripheral vascular disease: 34%, Atrial fibrillation: 11%	26%

Caregivers

Ten publications included information about caregivers [19-21, 30, 32, 33, 36, 41, 44, 46]. In all the studies, caregivers were informal, mainly (69.05%-93.5%) immediate family members, 61.2%-88.4% were women, 56%-70.1% were daughters/sons, and 26.7%-55.1% were partners [19, 32, 33, 36, 44, 46]. In many cases, the caregivers cohabited with the patients (56.7%-86.9%) [19, 20]. The education level of caregivers varied widely, with most having primary or secondary education (49.2%-75%), a smaller proportion having university studies (12%-29%; except one study reporting 85.8%), and a few having no formal education (3.4%-23%). Moreover, the reported percentage of caregivers with active employment was 21.8%-32.5% [19, 20, 41], except in one study conducted among employees of a pharmaceutical company [30].

Caregiver burden: Information about the caregiver burden was collected from six studies [19, 20, 30, 32, 36, 46]; five reported the time spent by the caregiver attending to the patient, ranging from 71.4 to 94.4 hours/week [19, 20, 30, 32, 46]. One article reported that most caregivers did full-time caring for the patient with AD [19], and three studies showed that the duration of caring varied between 40.9 and 79.1 months [19, 20, 36]. The caregiver's work/social life was partially or very affected in 42.9% and 14.3% of the cases, respectively [30]. Other data were only reported in individual studies, such as the treatment needed to cope with care (required by 33.7% of caregivers) [19], the use of medication due to caring tasks (in 50.3% of caregivers) [32], the mean free available time (10.7 ± 6.8 hours) [20], and the mean subjective burden (61.7% of caregivers reporting a heavy care burden score) [20]. None of the studies classify the caregiver burden according to AD severity.

Caregivers’ HRQoL: Caregivers’ mental health and ability to manage care situations were reported in twelve publications [19-21, 30-33, 36, 38, 41, 44, 46] (Table 9). Different HRQoL questionnaires showed that caregivers were in a below-average health status (30.3-49.5 Short Form-36 Health Survey Questionnaire (SF-36)) [19, 20, 41]. Indeed, questionnaires used to evaluate neuropsychiatric symptoms, mental health, and ability to manage care revealed an impact of caregiving in terms of anxiety or depression (40%-75%) [19-21], sleeping problems (28%-96%), and pain/discomfort (56%-95%) (Table 10).

**Table 9:** Caregiver HRQoL, mental health, and ability to manage care AD: Alzheimer’s disease; HRQoL: health-related quality of life; Alzheimer’s Disease Knowledge Scale (ADKS) is expressed as mean ± SD; the Beck Anxiety Inventory (BAI) score is expressed as N; the Beck Depression Inventory-Fast Screen (BDI-FS) score is expressed as N (%); The caregivers’ reaction scale is expressed as mean ± SD; the caregivers’ strain index score is expressed as mean ± SD; CASP-19 (quality of life questionnaire in older adults) is expressed as mean ± SD; The Connor-Davidson Resilience Scale (CD-RISC) is expressed as a mean ± SD; the CD-RISC-10 is expressed as a mean ± SD; CI 95%; The Coping Orientations to Problems Experienced (COPE28) Questionnaire is expressed as a mean ± SD; DUKE UNC-11 (Perceived Social Support Questionnaire) is as mean ± SD; EuroQol-5 Dimension (EQ-5D) is presented as a mean; The ESTE-II Scale is expressed as mean ± SD; the Goldberg Anxiety and Depression Scale (GADS) is expressed as mean ± SD; GC: accompanying control group; Goldberg's General Health Questionnaire (GHQ28) is expressed as mean ± SD; GI: psychotherapeutic group without active post-intervention follow-up; GIS: psychotherapeutic group with active post-intervention follow-up; Min-Max; MOS Social Support Survey (MOS-SSS) is expressed as a mean ± SD; Neuropsychiatric Inventory Questionnaire (NPI-Q) is expressed as mean (female-male) and N (%); The Orientation to Life Questionnaire (OQL-13) is expressed as a number of points; QoL: quality of life; the Revised Memory and Behavior Checklist (RMBC) is expressed as mean ± SD; the Rosenberg Self-Esteem Scale (RSES); the Short Form-36 Health Survey Questionnaire (SF-36) is expressed as mean ± SD; the Satisfaction Life Scale (SWLS) is expressed as mean ± SD; and the Zarit Burden Inventory (ZBI).

Author (year)	HRQoL questionnaire	Score
Casal Rodríguez (2019) [19]	EQ-SD	0.75
Durán-Gómez (2020) [20]	SF-36	30.3 ± 5.4 (Mean ± SD)
	ZBI	60.7 ± 13.7 (Mean ± SD)
	CD-RISC	69.2 ± 14.1 (Mean ± SD)
	MOS-SSS	74.3 ± 15.3 (Mean ± SD)
Garcia-Ribas (2020) [30]	SWLS	26.8 ± 5.6 (Mean ± SD)
	BDI-FS	1.6 (2.6) (N (%))
	ADKS	22.1 ± 2.9 (Mean ± SD)
	Caregivers’ reactions scale	26.8 ± 20.2; 0–74 (Mean ± SD; CI 95%)
	RMBPC	36.4 ± 25.7; 0–92 (Mean ± SD, CI95%)
Gomez-Gallego (2021) [31]	NPI symptoms	Delusions: 1.1–0.57, Hallucinations: 0.78–0.29, Agitation: 0.67–0.29, Depression: 1.11–0.57, Anxiety: 1.00–0.29, Elation: 0.44–0.29, Apathy: 0.43–0.43, Disinhibition: 0.56–0.86, Liability: 0.78–0.29 (Mean Female–Mean Male)
	NPI subscales	Mood: 2.89–1.14, Agitation: 2.67–1.43, Psychosis: 1.89–0.84, Frontal: 2.22–1.85 (Mean Female–Mean Male)
Gómez Maldonado (2023) [32]	Caregiver Strain Index	8.9 ± 3.1 (Mean ± SD)
Hernández-Padilla (2021) [33]	GHQ-28	30.8 ± 5.8 (Mean ± SD)
	Caregiver Strain Index	7.6 ± 3.2 (Mean ± SD)
	DUKE-UNC-11	35.7 ± 10.0 (Mean ± SD)
Luque-Carrillo (2020) [36]	ESTE-II scale	8.4 (4.3)
	BAI	11.1 (9.5) [N (%)]
	BDI-FS	8.3 (7.3) [N (%)]
	ZBI	29.2 (14.0) [N (%)]
Mariezcurrena (2020) [38]	COPE28	34.2 (8.0) [N (%)]
	ZBI	39.4 ± 14.2; 36.1–42.7 (Mean ± SD; CI95%)
	CD-RISC-10	24.7 ± 7.3; 23.0–26.3 (Mean ± SD, CI95%)
Poudevida (2022) [41]	SF-36	QoL, physical health (Mean ± SD): GI + GIS 52.4 ± 7.7 GC 50.9 ± 7.32 QoL, mental health (Mean ± SD): GI + GIS 49.5 ± 10.0 GC 48.7 ± 9.5 GI +GIS 52.4 ± 7.7 GC 50.9 ± 7.32
	CASP-19	GI + GIS: 38.2 ± 8.4 (Mean ± SD) GC: 37.9 ± 8.0 (Mean ± SD)
	GADS	GI + GIS: 3.3± 3.7 (Mean ± SD) GC: 2.3 ± 2.71 (Mean ± SD)
	ZBI	GI + GIS: 54.6 ± 14.0 (Mean ± SD) GC: 50.2 ± 13.0 (Mean ± SD)
	CD-RISC	GI + GIS 68.5 ± 13.6 (Mean ± SD) GC 67.3 ± 11.6 (Mean ± SD)
	DUKE-UNC-11	GI + GIS 39.3 ± 10 (Mean ± SD) GC 40.3 ± 8.6 (Mean ± SD)
Rosende-Roca (2022) [21]	NPI-Q score	Agitation/aggression: 122 (11.5%), Hallucinations: 95 (8.9%), Anxiety: 528 (49.6%), Apathy/indifference: 677 (63.6%), Aberrant motor behavior: 73 (6.9%), Delusions: 197 (18.5%), Depression/dysphoria: 429 (40.3%), Disinhibition: 72 (6.8%), Elation/euphoria, 14 (1.3%), Appetite and eating disorders: 154 (14.5%), Irritability/lability: 565 (53.1%), Sleep and night-time behaviors: 303 (28.5%) (N (%))
	ZBI	51.9 (Mean)
Ruiz-Fernández (2019) [44]	DUKE-UNC-11	AD-Early-Stage: 36.7 ± 10.0* (Mean ± SD) AD-Middle-Stage: 35.06 ± 10.0** (Mean ± SD) *p value <0.05 **p value <0.01
Turró-Garriga (2020) [46]	OQL-13	73.3 (Mean)

**Table 10:** Impact of caregiving for a patient with AD NA: not applicable/not available; AD: Alzheimer's disease

Author (year)	Symptoms reported by caregivers			
	Sleeping problems	Pain and related symptoms	Psychological	
			Behavioral	Emotional
Casal Rodríguez (2019) [19]	NA	Pain/discomfort: 56.8%	NA	Anxiety, depression: 72.8%
Durán-Gómez (2020) [20]	Sleep disorders: 95.8%	Pain: arms, legs, and joints: 95%, Back pain: 73.3%, Headaches: 72.5%, Fatigue: 90.9%, Gastrointestinal symptoms: Constipation or diarrhea: 68.4% Stomach pain: 57.5%	NA	Anxiety: 63.3%, Depression: 62.5%
Rosende-Roca (2022) [21]	Sleep and night-time behaviors: 28.5%	NA	Psychotic symptoms: Hallucinations: 8.9%, Delusions: 18.5%, Behavioral symptoms: Agitation/aggression: 11.5%, Disinhibition: 6.8%, Elation/euphoria: 1.3%, Irritability: 53.1%, Others: Appetite and eating disorders: 14.5%, Aberrant motor behavior: 6.9%	Apathy: 63.6%, Anxiety: 49.6%, Depression: 40.3%

Resource Utilization and Costs

Healthcare resource use: Eight publications reported data about the use of resources by patients with AD [25, 27, 28, 32, 34, 39, 43, 45, 46]. Most of the information regarding hospital resource use concerned admission rates [27, 34, 46], which increased as the disease progressed, as shown by Khandker et al. (regression analysis: mild AD: 0.2; moderate AD: 0.3; severe AD: 0.5) [34], and was independent of age and the presence of comorbidities. Additionally, Darbà and colleagues noted that 10.5% of all hospital admissions were due to dementia. The medical specialties accessed were internal medicine, neurology, geriatrics, and psychiatric services (46.2%, 19.1%, 10.8%, and 10.6%, respectively) [27]. Furthermore, Turró-Garriga and colleagues found that 19.1% of patients with AD sought emergency care, and 6.8% were hospitalized, with 70.1% attending outpatient consultations and 100% seeking specialist care over one year [46]. Finally, as reported by only one study evaluating patients with mild-to-moderate AD (GDS score of 4-6) who attended memory clinics, 15% and 14.9% of participants used domiciliary care and daycare centers, respectively [46]. No data were reported on the use of hospice care, palliative care, or other end-of-life services in the included studies, indicating a lack of evidence regarding service utilization at later disease stages. Regarding treatments and medical procedures for patients with AD, the mean number of prescribed medicines ranged from 5.8 to 7 [25, 39, 45] Two studies reported the prevalence of prescribed medication for AD: 52.6% of patients were prescribed acetylcholinesterase inhibitors in one study with patients with dementia institutionalized in residential care facilities [39]; in the other study, conducted in a regional healthcare area, 3.8% of patients aged 75-79 years and 7.9% of those aged ≥85 years received treatment [28].

Total costs: One recent national study estimated the total cost of direct healthcare, non-healthcare, and social care costs, as well as indirect costs, between December 2021 and February 2022, as €42,336.4-€70,445.1 per patient/year, depending on the method used to estimate non-healthcare costs. This study also reported that the direct cost per patient/year increased with AD severity. Using the proxy good method with the price per hour of a home employee (HE), the direct non-healthcare costs (mean ± SD) (including informal care cost) were: mild AD: €25,725.8 ± €26,116.8; moderate AD: €42,315.9 ± €24,329.3; and severe AD: €47,894.6 ± €34,896.7 [32]. However, the direct non-healthcare costs (mean ± SD) estimated using the price per hour of home help service (HHS) were higher: mild AD: €42,014.2 ± €42,677.1; moderate AD: €72,676.9 ± €45,683.4; and severe AD: €77,597.2 ± €59,260.2 [32]. There was no cost estimation for patients with MCI. This study reported that direct non-healthcare costs accounted for 84.6%-90.7% of total costs, followed by direct healthcare costs of 6.1%-10.0% and social care costs of 2.8%-4.6%. Indirect costs represented 0.5%-0.8% of total costs due to labor productivity losses resulting from reduced working hours or workdays because of the disease.

Direct healthcare costs: Two studies estimated the total direct cost per patient as €3,647.1-€4,969/year, evaluating data from 2011-2016 [27] and from December 2021 to February 2022 [32]; costs increased with disease progression (mean ± SD) (mild AD: €3,253.6 ± €7,349.8; moderate AD: €2,836.0 ± €2,840.4; severe AD: €4,623.2 ± €7,815.0) [32]. The main components of direct costs were hospitalizations, medical tests, visits to specialists, and emergency services. Extended hospital stays increased healthcare expenses from €1,040.7 [32] to €5,051 [27] per patient and year. Additionally, two studies showed that prolonged hospitalization also elevated costs in tests, specialist visits (mainly internal medicine services, €2,107.2 [46] to €5,531 [27] per patient and year), emergency services (€1,276.8 [46] to €5,057 [27] per patient and year), scheduled admissions, and readmissions (Table 11). Furthermore, one study reported increased direct healthcare costs mainly related to pharmacological costs (€862.7 per patient and year), outpatient visits (€644.3 per patient and year), palliative care (€489.2 per patient and year), and emergency services (€429.3 per patient and year) [32].

**Table 11:** Direct healthcare costs NA: not applicable/not available; €: Euros; Emergency services, scheduled admission, and readmission are expressed as mean (€). ‡Calculated data from the reported €/month and patient value in Turró-Garriga (2020) [47].

Direct healthcare costs	National: Darbà (2021) [27]	National: Gómez Maldonado (2023) [32]	Regional: Turró-Garriga (2020) [46]
Total direct healthcare costs (€ per patient and year)	4,818–5,393 (mean for study period: 4,969)	3,647.1 ± 6,086.3	NA
Outpatient visits (€ per patient and year)	NA	644.3 ± 598.6	NA
Patients deceased during the admission (€)	5,580 (≤ 7 days of hospital stay), 6,197 (> 7 days of hospital stay)	NA	NA
Medical care (€ per patient and year)	18,074 (≤ 7 days of hospital stay) 58,686 (> 7 days of hospital stay)	93.1 ± 234.5	NA
Pharmacological costs (€ per patient and year)	NA	862.7 ± 702.5	NA
Specialists	€ per patient and year: 17,895 (≤ 7 days of hospital stay), 19,175 (> 7 days of hospital stay)	NA	€ per patient and month: 175.6 (^‡^€ per patient and year: 2,107.2)
Emergency services	€ per patient and year: 4,653 (≤ 7 days of hospital stay), 5,057 (>7 days of hospital stay)	€ per patient and year: 429.3 ± 635.6	€ per patient and month: 106.4 (^‡^€ per patient and year: 1,276.8)
Hospitalizations	€ per patient and year: 4,626 (≤ 7 days of hospital stay), 5,051 (> 7 days of hospital stay)	€ per patient and year: 1,040.7 ± 3714.7	€ per patient and month: 2,067.7 (^‡^€ per patient and year: 24,812.4)
Palliative care (€ per patient and year)	NA	489.2 ± 2979.7	NA

Direct social care and non-healthcare costs. Social care, understood as non-medical support services that assist people with AD in daily living and maintaining independence, and non-healthcare costs are summarized in Table 12. The direct social care costs (mean ± SD) were estimated at €1,957.1 ± €2,815.5 per patient and year. These costs were associated with dependence and the psychological symptoms of patients with AD and ranged from €791.9 ± €1,444.7 per patient and year in mild AD to €2,372.8 ± €3,208.5 per patient and year in severe AD [32]. Direct non-healthcare costs (mean ± SD), presumably assumed by patient and family, were €36,364.8 ± €27,241.8 per patient and year (HE) and €64,473.5 ± €49,954.3 per patient and year (HHS). Direct non-healthcare costs also depended on AD progression and ranged from €21,680.0 ± €21,339.2 in mild AD to €40,026.2 ± €31,420.1 per patient and year in severe AD [32]. Most of the direct non-healthcare costs were due to informal care (€29,231.5 ± €24,350 per patient and year (HE), and €57,340 ± €47,765.4 per patient and year (HHS)), professional care, costs related to housing adaptation, and out-of-pocket expenses [32]. Additionally, two studies reported data about day centers, where costs ranged €1,758.9-€4,845.6 per patient and year [32, 46].

**Table 12:** Direct non-healthcare and social care costs per patient HE: When direct non-healthcare costs include informal care costs estimated with the proxy good method, using the price per hour of a HE as a proxy; HHS: when direct non-healthcare costs include informal care costs estimated with the proxy good method, using the price per hour of HHS. ‡ Calculated data from the reported €/month value in Turró-Garriga (2020) [47]. HE: home employee; HHS: home help service, NA: not applicable/not available; €: euros

Direct non-healthcare and social care costs		National: Gómez Maldonado (2023) [32]	Regional: Turró-Garriga (2020) [46]
Direct social care costs	Total direct social care costs (€/year)	1,957.1 ± 2,815.5	NA
	Nursing homes (€/year)	198.2 ± 1,245.4	NA
	Day-care centers (€/year)	1,758.9 ± 2,660.3	4,845.6
	Domiciliary care (€/month)	NA	322.5
	Other social care resources (€/month)	NA	164.4
Direct non-healthcare costs	Total Direct NON-healthcare costs (€/year)	36,364.8 ± 27,241.8^HE^ 64,473.5 ± 49,954.3^HHS^	NA
	Informal care (€/year)	29,231.5 ± 24,350.4^HE^ 57,340.1 ± 47,765.4^HHS^	NA
	Professional care (€/year)	3,450.5 ± 6,888.5^HE, HHS^	NA
	Caregiving time (€/month)	NA	842.8
	Housing adaptations (€/year)	1,653.5 ± 4,280.2^HE, HHS^	NA
	Orthopedic devices (€/year)	525.1 ± 1,288.6^ HE, HHS^	NA
	Out-of-pocket expenses (€/year)	1,278.9 ± 1,668.2^ HE, HHS^	NA
	Transportation (€/year)	225.3 ± 495.3^ HE, HHS^	NA
	Basic activities of daily living (€/month)	NA	420.5
	Instrumental activities of daily living (€/month)	NA	642.5

Indirect costs: Indirect costs (mean ± SD) at the national level related to labor productivity losses of patients with AD were estimated to be €367.4 ± €3386.8 (per patient and year) from a questionnaire aimed at informal caregivers [32]. This study included information on labor productivity losses due to the disease, as well as informal caregivers’ labor productivity losses due to caring for patients with AD. Patients cared for in this study had an average age of 79 years, with stages ranging from mild to severe AD dementia; MCI and early-onset AD were not included. Caregivers had an average age of 54-61 years. The earnings lost by the working patients due to sick leave or disease-related work absence were estimated as €15.32 and €17.08 per regular working hour for the women’s and men’s average wages, respectively. Indirect costs due to labor productivity loss also increased with disease severity and were €872.5 ± €5197.8 per patient per year in patients with severe AD [32]. Moreover, the earnings lost by the working patients because of reduced working hours or recurrent days of absence associated with AD were estimated using the national average hourly wage, by sex, employment type, and reduced or lost working hours. These losses were higher in men than women (average wage per year of €31,408.56 vs. €24,934.54, respectively). Additionally, the mean number of effective work hours per week was 43.0 and 38.0 hours/week for the self-employed men and women, respectively, and 37.7 and 33.3 hours/week for employed men and women, respectively.

Discussion

This systematic review provides a comprehensive overview of the epidemiology, diagnosis, patient journey, humanistic and caregiver’s burden, resource utilization, and costs of AD in Spain based on evidence published between January 2019 and January 2024. We included 26 studies with variable designs, disease stages, healthcare settings, and temporal and geographic scopes. Patients and caregivers included in these studies ranged from 69 to over 1,000,000 people, with a median size of approximately 150.

Incidence and Prevalence

Results suggest that current studies are insufficient to accurately determine the prevalence and incidence of AD in Spain. Evidence suggested significant underdiagnosis of MCI due to AD, particularly in primary care. In our SRL, the incidence of AD varies. This variability may be attributed to the period of analysis (1992-1996 vs. 2011-2016), the healthcare setting (medical reports or in-hospital incidence), and the geographical scope (national or regional). Increased life expectancy and changes in the diagnostic work-up could also explain this variability. The incidence reported from the SIDIAP database might be lower [40], as it was estimated in the general population with generic syndromic diagnoses. In contrast, other national studies included an older, institutionalized population presenting cognitive symptoms and multiple diseases [24, 27]. The observed incidence in the SIDIAP database (4.2 per 1,000 inhabitants in the population aged over 65) [40] contrasts with the average European incidence (11.08 per 1,000 inhabitants) [47]. This discrepancy may stem from an underdiagnosis of AD in Spain. Additionally, in individuals aged 80 and older, although cognitive impaired cases may be higher, etiological diagnosis often remains incomplete, which may be a result of few referrals to specialists for a definitive diagnosis, leading to a lack of clarity regarding the underlying cause of cognitive decline.

This review also highlights variability in AD prevalence reported in the included studies. The variability observed between prevalence estimates derived from large population-based databases and those reported by specialist-based registries suggests that epidemiological figures for AD are strongly shaped by the underlying data source and the diagnostic pathways used to identify cases [40, 47-49]. Population-based databases, particularly those drawing on administrative or primary care records, are useful for describing the overall burden of AD at the population level, but they are less likely to capture early-stage or etiologically defined cases. Specialist registries, by contrast, usually reflect more selected patient groups, most often assessed in memory clinics, where broader diagnostic workups are available and diagnostic certainty is consequently higher. These differences do not arise solely from methodological choices. They are also influenced by contextual factors such as socioeconomic status, educational level, and differences between rural and urban settings, as well as by unequal access to specialized services capable of completing full etiological assessments. Taken together, these factors affect both the timing of diagnosis and subsequent care pathways, including the use of residential facilities or day care services, which in turn increases the likelihood that individuals with AD are identified and recorded in healthcare databases [50-53].

The Spanish Society of Neurology (SEN) has highlighted the lack of adequate national records of cognitive impaired and AD cases and determined the epidemiology of AD and MCI due to AD in Spain, based on prevalence rates previously estimated by Gustavsson et al. (2023) [54]. According to these estimates, prevalence rates were 2.7% for ages 60-64, 3.8% for ages 65-69, and 5.2% for ages 70-74 [2]. In contrast, a recent study in the Basque Country, which focused on the validation of brief cognitive tests to diagnose MCI, reported a 23.10% prevalence of different CI stages, from MCI to dementia of any etiology, among individuals over 60. This finding reflects data from the general population rather than a specific cohort [55] and is much higher than the prevalence reported by the SEN [2]. Specifically, this population was assessed using the Cardiovascular Risk Factors, Aging, and Dementia (CAIDE) index ≥ 9, which reached 33.46%. Therefore, the information of this study reflects a population-level prevalence within a sample with an increased risk of cognitive impairment.

Most studies that were reviewed here included populations aged over 65. The mean age of patients with AD dementia is 70-80 years. The time to diagnosis ranges from six months to two years, and women are more affected than men [25, 30]. It should be noted that the first symptoms of MCI are often underrecognized, frequently misattributed to normal aging. Furthermore, there is a lack of data on the mean age at diagnosis, which would be valuable for understanding disease progression and early detection. The SEN has pointed out that progressive aging of the population and increased life expectancy will impact the number of cases of MCI and AD in the coming decades [7]. The development of better diagnostic practices and tools, including the refinement of brief cognitive screening tests, as well as the potential application of digital biomarkers, will also improve the detection of AD [56]. Currently, population-based studies on AD are limited and mainly conducted in geriatric centers, where advanced stages are more prevalent [25, 26, 34, 46]. This limits our understanding of the true prevalence of AD in Spain, particularly in its early stages. The scarce information makes it harder to study the early stages of the disease, which in turn delays progress in early diagnosis and the development of treatment strategies.

Mortality

In 2023, the Spanish National Statistics Institute estimated 21,084 deaths due to dementia (representing the fourth most common cause of death) and 13,172 deaths due to AD in Spain (3.03% of the total number of deaths, the seventh cause of death) [57]. These figures likely underestimate the true mortality, as AD and dementia are often not recorded as the primary cause of death. Mortality data were very scarce in the studies analyzed in this review; only one study reported the mortality rate (9.5%), which was related to hospitalizations [27]. In this study, Darbà and colleagues observed that mortality was mainly due to respiratory problems and heart failure [27]. However, determining the immediate cause of death may be subject to bias, as respiratory infections are common in clinical contexts, and heart failure is often an outcome induced by other underlying factors. Moreover, the studies included in this SRL suggest that AD usually coexists with other comorbidities, such as cardiovascular risk factors and cerebrovascular diseases [19, 20, 24-27, 34, 40, 45], which may directly impact survival and/or worsen dependency.

Diagnosis

The predominance of moderate-to-severe AD dementia suggests a delayed diagnosis, which results in a lower detection of MCI. The variability in the study settings, mainly conducted in residential care facilities, does not capture the full spectrum of AD dementia, which highlights the lack of an adequate national registry for such data. Currently, the diagnosis of AD dementia is a sequential process, from syndromic diagnosis, based on semiology and findings in neurological-neuropsychological examination, to the determination of the specific biological fingerprint of some diseases such as AD [2]. However, in Spain, there is considerable variability in diagnostic and treatment processes across regions, and diagnostic tests are not uniformly available [58]. This SLR reveals that the MMSE test is the most frequently used diagnostic tool despite its suboptimal performance in detecting MCI and identifying atypical presentations of AD dementia in initial dementia stages [59]. The techniques most frequently used for etiological diagnosis were structural neuroimaging techniques, such as computed tomography and MRI, along with general blood tests [43, 46].

The low use of CSF biomarkers and PET in the studies included in this SRL may be due to the inclusion of subjects at more advanced stages, where these approaches are not clinically indicated in routine care [2, 58, 60]. This underscores the need for better diagnostic processes, transitioning from syndromic to etiological diagnosis, especially in early stages. Early diagnosis and monitoring with these tests could help slow AD by detecting molecular changes and targeting disrupted pathways [61]. In this context, the future applicability of plasma biomarkers offers a promising alternative that will facilitate the early diagnosis of AD [2, 62, 63]. In this regard, p-Tau217 has demonstrated a high diagnostic accuracy with precise individual prediction in a real-world clinic cohort [29, 42]. Additionally, once DMTs become available, APOE genotyping will be necessary for eligible individuals, despite not currently being recommended in routine clinical practice [64].

Patient Journey

Information about the patient journey is also scarce. Neurologists are the specialists primarily responsible for the diagnosis of AD, which spans several months to more than two years from symptom onset [30, 32]. However, most of the studies have included populations in late stages of dementia, and early dementia symptoms may have been misattributed to age-related cognitive decline. Other specialists involved in AD diagnosis included primary care physicians, geriatricians, and psychiatrists [30, 43]. A systematic review found barriers along the patient journey, such as resistance to seeking care among patients with AD [65]. Thus, promoting studies focusing on the outcomes and experiences reported by patients with AD in Spain could be valuable in helping improve the patient journey.

Caregivers

Most caregivers of patients with AD are informal, primarily women (more than 50%) [21], immediate family members, and cohabit with the patients [19, 20]. The burden of caregiving impacts multiple health dimensions. Caregivers face considerable emotional and physical strain, mainly anxiety and depression [19-21]. Disrupted sleep, highlighted in several studies, directly impacts caregivers' quality of life and is a modifiable risk factor for multiple diseases [20, 21]. A recent systematic review evaluated the interventions that could reduce caregiver burden in various dementia diseases; the study suggested that individual therapy ameliorates caregivers’ stress, which can be further improved in group therapy by enabling interactions with others in the same situation [66]. Therefore, there is a need to improve support systems for caregivers, including advisory services and psychological support [58].

Healthcare Resources and Costs

Most information on healthcare resource utilization indicates that service utilization, particularly hospital admissions, increases as AD progresses, reflecting increasing clinical complexity at older ages [27, 34, 46]. Across studies, non-healthcare and social care needs emerged as major drivers of overall costs, largely due to the burden of dependence, behavioral symptoms, and the extensive reliance on informal caregiving [32]. Healthcare costs were mainly associated with hospital care, diagnostic procedures, specialist visits, and emergency services [27, 32, 46], while additional support, such as domiciliary or day care services, was used by only a subset of patients [46]. Indirect costs related to lost productivity for both patients and caregivers also contributed meaningfully to the overall economic burden [32]. This is in line with recent data showing that 80% of the total direct non-healthcare costs and total costs of AD are due to informal care and non-medical direct costs, and are significantly higher in moderate-to-severe AD dementia [67]. Additionally, approximately 3%-4% of adults aged ≥65 years in Spain remain employed according to the National Institute of Statistics, reflecting the limited contribution to the socioeconomic burden of the disease [68]. Thus, early diagnosis and treatment could contribute to a better organization of resources and optimization of care, which could reduce the economic impact on the healthcare system, patients, and their families/caregivers. Additionally, multimodal non-pharmacological interventions, supported by the Dependency Law [69] or the FINGER program [70], which focus on lifestyle-based interventions to reduce the risk of CI or dementia, may contribute to slowing cognitive decline. These interventions could be integrated into the universal healthcare system so that patients can access DMTs and improve their outcomes.

Limitations

Our review has some limitations. First, although the search strategy was designed to identify studies most relevant to the Spanish context, the exclusion of some international databases such as the Excerpta Medica database (Embase) or Scopus may have limited the retrieval of additional publications indexed exclusively in broader bibliographic sources. In addition, there is considerable heterogeneity in the studies included in this SLR in terms of population characteristics and study designs, which hinders the synthesis of the results and their interpretation. Most studies evaluated populations aged 65 and older from nursing and geriatric centers, or cohorts already diagnosed with dementia, revealing a gap in the real epidemiology of AD in Spain and the disease’s onset in younger individuals.

Furthermore, the heterogeneity of study designs, populations, and outcome measures limited the feasibility of a quantitative synthesis, and findings were therefore integrated using a structured narrative approach.

Evidence presented as abstracts has been omitted from our review and may have resulted in the omission of potentially relevant data. Future research should focus on pre-dementia stages; studies that include all forms of late-onset, sporadic, or genetically determined AD; caregiver support; and optimization of healthcare resources to address the growing impact of AD in Spain effectively.

Part of the study period coincided with the COVID-19 pandemic, which may have affected healthcare delivery, access to diagnostic services, and reporting practices in some of the included studies. At the same time, the data reflect routine clinical practice under constrained healthcare conditions and offer insight into how diagnostic pathways and care processes for AD were maintained during a period of substantial system-level disruption.

## Conclusions

This SLR underscores the urgent need to advance early diagnosis of AD dementia and to establish reliable health registries to accurately determine its true extent in Spain. The lack of national strategies for a comprehensive understanding of the continuum of AD hinders effective management and planning of care for individuals with different stages of the disease. Reliable health registries are essential for better resource allocation and informed decision-making, especially given the growing number of cases, driven by an aging population.

The study underscores the existing heterogeneity in access to early and accurate diagnosis across Spain. Variability in diagnostic practices, tools, and regional resources contributes to delays in AD diagnosis, particularly in its early stages, when timely intervention could have the most significant impact. Addressing these disparities is crucial to ensuring that all individuals, regardless of their location, can access early and effective care, especially with the advent of DMTs. Although the Spanish National Healthcare System provides universal coverage, the economic burden of diagnosing and managing AD dementia remains substantial, placing considerable financial strain on both the healthcare system and families/caregivers of patients with AD. This burden is further exacerbated by the increasing emotional and social challenges faced by caregivers. Addressing these issues is vital for improving the management of patients with AD in Spain, enhancing their HRQoL, and ensuring that families/caregivers receive the necessary support, while also mitigating the significant economic impact on both families/caregivers and the healthcare system.
